# Book Review

**Published:** 1934

**Authors:** 


### Reviews of Books

**The Physician's Art.** By A. G. Gibson. Pp. viii., 237. Oxford: Clarendon Press. 1933. Price 7s. 6d.—The greater part of this delightful little book consists of aphorisms and discussions on the work and life of an ideal physician, reminding one much of Osler's Æquanimitas. The sections on Diagnosis, Prognosis and Treatment are full of invaluable suggestions and warnings, culled from a wide experience of men and of disease. Equally important are the pages on medical ethics and on the doctor's care of himself. The initial chapter has an interest of its own, for it contains a reprint of John Locke's *De Arte Medicâ*, 1669, an inquiry as to the method by which medicine can be best improved. This was a very urgent question at that time, for it was clear that the old logical method by framing hypotheses and making deductions was useless, and Locke's great friend and teacher, Sydenham, had decided against the plan of building up medicine by the laws of the new physical sciences, and fell back on the Hippocratic method. In this paper Locke seems to have struggled in vain to reach a solution, but it is significant that the very next year he found that human thoughts and words were so loose and uncertain that he turned himself to psychology and began his immortal *Essay on the Human Understanding*.

**Behind the Doctor.** By Logan Clendening. Pp. xxi., 458, xi. Illustrated. London: William Heinemann Ltd. 1933. Price 21s.—This is a history of medicine of a new type, by an American writer, and primarily intended for the lay reader. The style is clear and attractive, and the author shows remarkable mastery of his subject and a marvellous industry in the collection of facts which were scattered over a wide literature. A large part of the book, after a rather sketchy account of primitive magic, is taken up with brilliantly-written conversations by or interviews with the discoverers and great teachers of every age. Now these stories are many of them perfectly authentic or, where they are imaginary, they expound the man's teaching as faithfully as possible.

Thus we have an interview between Galen and his patient, the Emperor Marcus Aurelius, another between Sydenham and young Hans Sloane, both of which actually took place; while there is actual evidence for the story of the discoverer of morphine being nearly poisoned by it, and for the picture of Addison and Richard Bright going round their wards teaching their students. The author inquires where is the statue to Sir Humphry Davy, Bristol's discoverer of "laughing gas"; it stands, of course, looking down the main street of Penzance. Very rarely can any inaccuracy be found in the account, though the misprint "forcibly interred" (p. 239) for "interned" is somewhat startling. And in the comparison between inoculation and vaccination on page 217 there should be a definite clause added that the former creates new centres of infection from which the disease may easily spread, whereas the latter does not: without this the account is misleading. But, taking the book as a whole, it is a lively, instructive and entertaining account of the development of medical science up to the present time, with numerous amusing biographical details, illustrations and portraits that will appeal probably even more to the medical man than to the layman.

**Applied Physiology.** Fifth Edition. By SAMSON WRIGHT, M.D. Pp. xxxii., 604. Illustrated. London: Oxford University Press. 1934. Price 18s.—In the new edition of this valuable text-book the whole matter has been thoroughly revised and brought up to date. This has necessitated a certain amount of rearrangement of the material, and many new illustrations have been added. Happily the book has thereby been but little increased in size, thanks to judicious pruning and to the deletion of obsolete material. It would appear, however, that clinical applicability is not always the main factor influencing the selection of matter. For instance, there is no clear account of the physiology of sight or hearing, which has been omitted, according to the Preface, to economize space, whereas several pages are devoted to the neck and otolith righting reflexes; but possibly these are important to the student of physiology if not to the clinician. While the book is perhaps not quite suitable for the beginner, it will continue to be indispensable both to clinical students and to practitioners anxious to keep abreast with the advances of physiology, and especially to those reading for higher medical examinations.

**A Text-book of Medicine.** Fourth Edition. Edited by FREDERICK W. PRICE, M.D. Pp. xlv., 1,995. Illustrated. London: Oxford University Press. 1933. Price 36s.—In the new edition of this popular text-book the whole subject-matter has been thoroughly revised, many articles having been rewritten and some new articles incorporated. Unfortunately, this has led to a further slight increase in the size of the book. Although thin paper is used in this, as in previous editions, the poor quality of the print noted in our review of the third edition has been considerably improved. The majority of the articles are excellent, and maintain the high standard set in the previous editions. A typical example of the way in which the work has been brought up to date is the article on "Erythema Nodosum," by Lord Horder. The illustrations are clear and well reproduced, but there appears to be rather a preponderance of pulse tracings and electro-cardiograms. This edition will doubtless retain and increase the popularity earned by its predecessors among both students and practitioners of medicine.

**The Chances of Morbid Inheritance.** Edited by C. P. BLACKER, M.D. Pp. xi., 449. Illustrated. London: H. K. Lewis & Co. Ltd. 1934. Price 15s.—This appears to be the first serious attempt, by a series of specialists, to put before the general practitioner a synopsis of the knowledge necessary to deal with the not infrequent requests made by the public for eugenic prognosis. The intelligent layman is definitely beginning seriously to consider the effect of sound and unsound mating, and his appeal is to the medical profession; the time has come when the latter can no longer consider a knowledge of eugenics as relatively unimportant, instead of being, as it is, the keystone to the ultimate success of medicine in its prevention and handling of disease and hereditary defects. The space devoted to each subject has been in proportion to the frequency of demands for guidance from the general practitioner by the layman, rather than to the extent of the knowledge acquired of hereditary influence in a particular disorder. Eugenics has two sides, the positive, that is the furthering of the propagation of sound stock, and the negative, the prevention of the multiplication of those with bad heredity. The negative side is particularly dealt with in this book, that with which the man in general practice is constantly in contact, and with which an increasing demand will be made on his knowledge of the subject, for the public is being steadily educated to its importance. The articles are well abreast of present knowledge, and deal lucidly and impartially with the subject so far as our present knowledge goes, although a better grasp would be acquired of what is here set out if the reader unacquainted with the problems of heredity would first study such a brief outline as Pannett's *Heredity*. Numerous references will be found for those who wish to extend their knowledge of the subject.

**Clinical Investigation of Cardiovascular Function.** By V. PACHON and R. FABRE. Translation by J. F. HALLS DALLY, M.D. Pp. xi., 252. Illustrated. London: Kegan Paul, Trench, Trubner & Co. Ltd. 1934. Price 15s.—This book is published in a series known as the "Anglo-French Library of Medical and Biological Science," the object of which is to promote greater interchange of scientific thought between the two countries. Monographs written by authorities on the subject in France are translated and published in English, and *vice versa*. The present book deals with the various methods of investigating the functions of the cardiovascular system, and includes detailed consideration of direct cardiography and the various uses of oscillometry. It is particularly interesting to read the views of the originator of these two methods of clinical investigation, especially in view of the increased importance which oscillometry is assuming in the study of peripheral vascular disease. Although English clinicians do not attach so much value to some of these methods as do the authors, the book is of considerable value in giving a clear and concise exposition of these special features of French cardiology. The translation is well done, the type clear and easily read, and the illustrations ample.

**Vitamins and Other Dietary Essentials.** By W. R. AYKROYD, M.D. Pp. x., 218. London: William Heinemann Ltd. 1933. Price 7s. 6d.—"Aykroyd on Vitamins" is the standard work on the subject. He has given a brief and readable account of the vitamins and the effects of their presence or absence from human dietaries. Beyond this he has presented in his book a short but comprehensive survery of the practical problems of nutrition. He drives home his observations with a humour somewhat reminiscent of Sydney Smith. "Under-nourished children," he writes, "are apathetic, do what they are told and are generally all that an adult can desire of children." Or elsewhere, discussing the investigations into the causation of pellagra, he mentions that a number of commissions appointed by the United States Government had failed to make headway with the problem. "No real discovery," he concluded, "is ever made by commissions." Many excellent books have been written on "Nutrition," here is incomparably the best one on 'Malnutrition."

**Essays on Chronic and Familial Syphilis.** By GRIFFITH EVANS, D.M. Pp. 91. Illustrated. Bristol: John Wright & Sons Ltd. 1934. Price 7s. 6d.—The latent form of syphilis to which the author introduces us is not ordinary syphilis which has passed into a latent stage, but a disease without chancre, exanthema, gumma, ulcer or Wassermann reaction; transmissible in the same "latent" condition, and to the third generation; characterized pathologically (as far as can be discovered from a far from clear account) by paravertebral lymphadenitis, "which must necessarily by proximity" affect the adjacent visceral sympathetic nerves, and clinically responsible for an enormous mass (or ought we to say the whole ?) of chronic disease. The importance of the therapeutic test has been much exaggerated: if every chronic patient who received benefit from a course of mercury or iodide is syphilitic the disease is indeed almost universal. A large part of the book is devoted to disease of the alimentary tract: nearly every form of chronic glossitis is clear evidence of syphilis. Achalasia and dysphagia with anæmia are considered as one disease, which suggests a very superficial knowledge of even the symptomatology of these conditions. The author repeatedly sneers at "teaching hospitals," implying that the staffs regularly neglect family history and clinical signs: "The routine procedure in hospital practice is to perform a Wassermann reaction, and to dismiss the diagnosis of syphilis when the result is negative," is so grossly untrue that one can only excuse it on the ground of careless proof-reading. It is, of course, true that a responsible member of a hospital staff requires something more than a vague hypothesis before making a diagnosis; he would never accept the "cancer family," of which both parents and five out of six adult children "died of cancer of the stomach," without at least some pathological confirmation. From the title of this book we hoped that it was a clear, well-reasoned reminder that the physician must be ever on the watch for the protean manifestations of syphilis ; as it is, exaggeration and a complete absence of logical and critical faculties leave the reader irritated but unconvinced.

**Venereal Disease.** Fifth Edition. By HUGH WANSEY BAYLY. Pp. xv., 260. Illustrated. London : Chapman & Hall Ltd. 1934. Price 10s. 6d.—This is the most outspoken and stimulating book on this subject that has been presented for a long time. The author makes bold statements that will arrest attention, especially in the section of the book that deals with prevention and with the place and prevention of venereal disease in modern life. His plea for standardization of the methods of diagnosis and treatment is sound. Syphilis is dealt with in a very comprehensive manner in over a hundred pages ; in particular, the primary stage is well described, which should be a boon to students of to-day, who do not see so many Hunterian chancres as did their predecessors. The methods of treatment are carefully described, as are the uses of the microscope and Wassermann reaction in diagnosis, and the need for examination of the cerebro-spinal fluid in some cases before accepting the patient as cured. Gonorrhœa receives adequate attention without much matter that is new ; the uses of the urethroscope might have had greater prominence. Certain other conditions that may be confused with venereal infection, for example genital herpes, are also carefully described, a point of considerable importance for those without much practical experience. The plates are extremely interesting and well produced. The many new features of this edition will be welcomed by students whose requirements are always kept in mind.

**A Synopsis of Public Health.** By E. W. CARYL THOMAS, M.D., B.Sc., D.P.H. Pp. vii., 646. Bristol : John Wright & Sons Ltd. 1932. Price 21s.—Dr. E. W. Caryl Thomas, Medical Officer of Health, Dagenham, has added another volume to the well-known " Synopsis " Series published by John Wright & Sons. This book deals with " Public Health " according to the curriculum for the D.P.H. Of its twenty-two chapters the first six deal with epidemic and communicable diseases and the various enactments dealing with them. The descriptions of the diseases are so admirably summarized that one might recommend the book to practitioners for this feature alone. Deficiency diseases, on the other hand, are by no means adequately described, although many of them are mentioned in the chapter on "Food and Dietetics." The same criticism might apply to some of the occupational diseases, for instance, silicosis. It would have been of greater value perhaps to have given more space to the clinical manifestations of these less familiar conditions and economized on the "fevers." Apart from this there is nothing but praise to be given to a book whose compilation must have been most difficult. The author has been most successful in blending the medical, bacteriological and chemical aspects of his various topics with the law in connection with them. As an instance, section 3 of chapter xiii may be referred to, which compresses into four pages the subject of "Schools and Infectious Diseases." Not only is a tremendous lot of information condensed into mininal space, but the section still remains readable. In part this readableness of the book depends on clever variation in type, in an even greater measure it depends on the choice of clear, short words in well-constructed sentences.

**Practical Bacteriology.** Fourth Edition. By T. J. MACKIE, M.D., and J. E. MCCARTNEY, M.D., D.Sc. Pp. viii., 504. Illustrated. Edinburgh: E. & S. Livingstone. 1934. Price 12s. 6d.—The fourth edition of this popular practical handbook maintains the high level of its predecessors. The subject, matter is now divided into three clear sections: biology and immunity; technique; diagnosis of infections with descriptions of pathogenic and commensal micro-organisms and viruses. Directions for senior and junior laboratory workers are also subdivided. The book thus offers a comprehensive survey of the whole subject, well arranged and concisely stated. The index is admirably set out and easy for reference. Authors and publisher may alike be congratulated upon a highly valuable issue.

**Massage and Remedial Gymnastics.** Third Edition. By LOUISA L. DESPARD. Pp. xxiv., 474. Illustrated. London: Oxford University Press. 1932. Price 22s. 6d.—The third edition of this standard text-book of massage is worthy of the high place secured by its predecessors. It is not intended as a means of instruction in massage: indeed, as the author points out, massage can be learnt only by actual practice under a competent teacher. But to enable the student to understand the principles underlying the actual manipulations such a work as this is essential, and it is difficult to see how it could be better supplied. A full account is given of the anatomy, with numerous coloured illustrations, and clear explanations of the normal functions of muscles, ligaments, bones and nerves, 220 pages in all. Next comes a section describing Swedish remedial exercises and the various procedures that are classed together as "massage," with detailed instruction as to how each measure is applied, what conditions may be thus treated, and what special precautions are needed in particular diseases or with certain patients. The latter part of the book is devoted to electrical methods, including galvanism, faradism, diathermy and electrical baths. There is a very complete index, the book is well printed on good paper, and we must congratulate both author and publisher on the production of a book that every student of massage, masseuse and the practitioner who contemplates treatment of his patients by these essential methods will find complete and indispensable.

**Hydrotherapy and Physiotherapy.** By LIONEL C. E. CALTHROP, M.B. Pp. 172. Illustrated. London: William Heinemann Ltd. 1931. Price 5s.—This little manual is designed as a guide for the bath attendant who actually administers the particular bath prescribed by the physician at a spa. In it are described the features of various forms of bath, pack, douche, etc., in simple, non-technical language. Careful directions are given as to the method of receiving a patient "who may be tired, irritable or in pain as a consequence of his disease," precautions to observe in arranging the bath or other treatment, faults to avoid, and a host of other valuable tips—we hope, enough to lead to tips of another description. Some of the statements are rather irritating: "Water is a clear liquid, colourless, tasteless and inodorous: in thick layers it has a blue tint." But if we disregard a tendency to obviousness, we have here a book well calculated to serve the reader to whom it is addressed. It is well illustrated, and contains a short, but sufficient, index.

